# Colonization of Onions by Endophytic Fungi and Their Impacts on the Biology of *Thrips tabaci*

**DOI:** 10.1371/journal.pone.0108242

**Published:** 2014-09-25

**Authors:** Alexander M. Muvea, Rainer Meyhöfer, Sevgan Subramanian, Hans-Michael Poehling, Sunday Ekesi, Nguya K. Maniania

**Affiliations:** 1 Institute of Horticultural Production Systems, Section Phytomedicine, Leibniz Universität Hannover, Hannover, Germany; 2 Plant Health Division, IPM cluster, International Centre of Insect Physiology and Ecology, Nairobi, Kenya; South China Agricultural University, China

## Abstract

Endophytic fungi, which live within host plant tissues without causing any visible symptom of infection, are important mutualists that mediate plant–herbivore interactions. *Thrips tabaci* (Lindeman) is one of the key pests of onion, *Allium cepa* L., an economically important agricultural crop cultivated worldwide. However, information on endophyte colonization of onions, and their impacts on the biology of thrips feeding on them, is lacking. We tested the colonization of onion plants by selected fungal endophyte isolates using two inoculation methods. The effects of inoculated endophytes on *T. tabaci* infesting onion were also examined. Seven fungal endophytes used in our study were able to colonize onion plants either by the seed or seedling inoculation methods. Seed inoculation resulted in 1.47 times higher mean percentage post-inoculation recovery of all the endophytes tested as compared to seedling inoculation. Fewer thrips were observed on plants inoculated with *Clonostachys rosea* ICIPE 707, *Trichoderma asperellum* M2RT4, *Trichoderma atroviride* ICIPE 710, *Trichoderma harzianum* 709, *Hypocrea lixii* F3ST1 and *Fusarium* sp. ICIPE 712 isolates as compared to those inoculated with *Fusarium* sp. ICIPE 717 and the control treatments. Onion plants colonized by *C. rosea* ICIPE 707, *T. asperellum* M2RT4, *T. atroviride* ICIPE 710 and *H. lixii* F3ST1 had significantly lower feeding punctures as compared to the other treatments. Among the isolates tested, the lowest numbers of eggs were laid by *T. tabaci* on *H. lixii* F3ST1 and *C. rosea* ICIPE 707 inoculated plants. These results extend the knowledge on colonization of onions by fungal endophytes and their effects on *Thrips tabaci*.

## Introduction

In Kenya, onions *Allium cepa* L. (Asparagales: Amaryllidaceae), are grown in all regions by both large- and small-scale farmers, where they have a ready domestic and regional market [1]. Onion thrips, *Thrips tabaci* Lindeman (Thysanoptera: Thripidae), is considered the most economically important pest of onion worldwide [2], [3]. In Kenya, it is present in all onion growing areas and can cause up to 59% loss in yield [4]. Currently, growers manage thrips by applying insecticides which are ineffective due to the cryptic feeding behavior of thrips, overlapping generations and insecticide resistance [5], [6]. Therefore, an integrated approach that includes the use of entomopathogens, cultural practices, host plant resistance and judicious use of insecticides is needed [7], [8].

Entomopathogenic fungi (EPF) are considered as important biocontrol agents (BCAs). They are traditionally applied in an inundative approach [9], but recent studies have shown that EPF play diverse roles in nature including as endophytes [10]. Indeed, the endophytic niche in a plant is a rich source of microorganisms that can directly and indirectly promote plant growth and development through plant defence against herbivorous insects [11] and plant pathogens [12], [13] due to their ability to produce secondary metabolites with biocidal activity [14], [15]. On a wide variety of crops, fungal endophytes have been reported to deter feeding, oviposition and performance of stem boring, sap sucking, chewing, and leaf mining insects [11], [16], [17], [18]. For example, endophytic colonization of banana by *Beauveria bassiana* significantly reduced larval survivorship of banana weevil, *Cosmopolites sordidus* (Coleoptera: Curculionidae), resulting in 42–87% reduction in plant damage [19]. Reduction in feeding and reproduction by *Aphis gossypii* (Hemiptera: Aphididae) has also been reported on cotton endophytically colonized by either *B. bassiana* or *Lecanicillium lecanii* (Hypocreales: Clavicipitaceae) [20].

Advantages of the application of endophytes over conventional foliar application of fungal entomopathogens [7] [21] are the ability to colonize plants systemically, thereby offering continuous protection and enhanced persistence [22]. Moreover, considerably low inoculum is required when applied as seed treatment [23]. However, colonization of a host plant by an endophyte is influenced by the inoculation method, species of fungal endophytes and the host plant species itself. Based on the inoculation technique, the endophytes differ in their ability to colonize different plant parts and to persist over a crop growth cycle [24], [25], [26]. Akello et al. [25] reported a higher colonization of tissue-cultured banana by *B. bassiana* through dipping roots and rhizome in a conidial suspension as compared to injecting a conidial suspension into the plant rhizome and by growing the plants in sterile soil mixed with *B. bassiana*-colonized rice substrate. Bing and Lewis [24] reported improved colonization of corn plants through foliar spray of conidia as compared to injection of conidia suspension. They also demonstrated the ability of *B. bassiana* to invade maize plants through the epidermis, thereafter persisting in the plant through the entire growing season, which conferred crop resistance against damage by European corn borer. However, information on endophyte colonization of onions and impact of endophytes on thrips infesting onions is not available. Hence, this study aimed to evaluate the efficacy of two procedures, seed and seedling inoculation methods, on colonization of onion plants by fungal endophytes and further assess their impact on infestation by onion thrips, and on thrips feeding and oviposition. Post-inoculation recovery of all the endophytes tested wase highest with seed inoculation method compared to seedling inoculation method, and this method was selected for the additional impact studies with thrips.

## Materials and Methods

### Ethics statement

The study was not undertaken in a national park or any other protected areas of land. The plants (onion), endophytes and the insect pest (thrips) involved in the study are not endangered or protected species. No specific permits were required to undertake the field studies in the locations mentioned. However, we obtained prior permission from the farmers in whose fields the sampling was undertaken.

### Biological material

#### Fungal isolates

Five fungal isolates (*Clonostachys rosea* ICIPE 707, *Trichoderma atroviride* ICIPE 710, *Trichoderma harzianum* ICIPE 709, *Fusarium* sp. ICIPE 712, and *Fusarium* sp. ICIPE 717 with GenBank Accession Nos: KJ619987, KJ619990, KJ619989, KJ619992 and KJ619993, respectively) were used in this study. The endophytes were isolated from onion plants asymptomatic of any pathogenic infection, collected during a field survey conducted in different altitudinal gradients of Kenya, namely Nakuru (00.01 N 36.26 E, 2000 m.a.s.l.), Loitokitok (02.71 S 37.53 E, 1200 m.a.s.l.) and Kibwezi (02.25 S 38.08 E, 825 m.a.s.l.) as detailed in the GenBank Accessions mentioned above. Two fungal isolates (*Hypocrea lixii* F3ST1 and *Trichoderma asperellum* M2RT4) isolated from the aboveground parts of maize and sorghum, and previously reported endophytic on maize and bean seedlings [27], were also included. Conidia were obtained from two-week-old cultures grown on potato dextrose agar (PDA) plates. The conidia were harvested by scraping the surface of sporulating cultures with a sterile scalpel. The harvested conidia were then placed in universal bottles with 10 ml sterile distilled water containing 0.05% Triton X-100 and vortexed for 5 min to produce homogenous conidial suspensions. The conidial concentration was determined using Neubauer hemocytometer. The conidial concentration was adjusted to 1×10^8^ conidia mL^−1^ through dilution prior to inoculation of seeds and seedlings.

To assess the viability of the conidia, 100 µL of conidial suspension was inoculated to the surface of two fresh plates of PDA for each isolate. A sterile microscope cover slip (2×2 cm) was placed on top of the agar in each plate before incubation. The inoculated plates were incubated for 24 h at 20°C. The percentage conidial germination was assessed by counting the number of germinated conidia out of 100 in one randomly selected field. Conidia were considered as germinated when germ tubes exceeded half of the diameter of the conidium. The percent germination of the different isolates exceeded 90%, which is recommended by Parsa et al. [28].

#### Insects

Initial cultures of T. tabaci were field-collected from onion plants at the International Centre of Insect Physiology and Ecology (icipe) organic farm. Thrips were reared on snow peas, Pisum sativum L. (Fabales: Fabaceae), for over 30 generations in ventilated plastic jars at the icipe’s insectary at 25±1°C, 50–60% relative humidity (RH), 12 h L: 12 h D photoperiod.

#### Onion seeds

Onion can be established using either direct seed sowing or seedling transplanting [29]. Seeds of onion (var. Red Creole) were surface-sterilized in 70% ethanol and then immersed in 2% NaOCl (bleach) for 2 and 3 min, respectively. The seeds were finally rinsed three times using sterile distilled water to ensure epiphytes were not carried on the seed surface. To confirm the efficiency of the surface sterilization methods, 100 µl of the last rinse water [28], [30] was spread onto potato dextrose agar and plates were incubated at 20°C for 14 days. The absence of fungal growth on the medium confirmed the reliability of the sterilization procedure. The seeds were then placed on sterile filter paper to dry for 20 min before being divided into two portions, one for the seed and the other for the seedling inoculation.

### Seed and seedling colonization of onion plants by fungal endophytes

#### Seed inoculation of fungal endophytes

For seed inoculation, 10****g of surface-sterilized seeds were subdivided into eight equal portions whereby seven portions were individually soaked in a conidial suspension of 1×10^8^ conidia ml^−1^ of each isolate for 10 hours. In the control, the eighth portion was soaked in sterile distilled water containing 0.05% Triton X-100. The inoculated seeds were air dried on a sterile paper towel for 20****min and then transferred in plastic pots (8****cm diameter×7.5****cm height) containing sterile planting substrate. The substrate was a mixture of red soil and livestock manure in a 5∶1 ratio and was sterilized in an autoclave for 2****hr at 121°C and allowed to cool up to ambient temperature before being used. Seeds were sown 1****cm below the surface of the substrate and maintained at room temperature (∼25°C and 60% RH) in the screen house. After germination, seedlings were thinned to one per pot for all the eight treatments and the four replicates. The plants were watered once per day in the evening. No additional fertilizer was added to the planting substrate.

#### Seedling inoculation of fungal endophytes

For seedling inoculation, surface-sterilized seeds, as described earlier, were raised in a plastic bucket (30 cm diameter×28 cm height) with sterile planting substrate and maintained in a screenhouse at room temperature (∼25°C and 60% RH) for one month before transplanting. Before transplanting, seedlings (height 7–8 cm) were watered and uprooted carefully to minimize damage to roots. After uprooting, the plants were shaken gently to dislodge excess soil on the roots, which were further washed with running tap water. Roots of four seedlings with well-developed shoots were dipped in each of the seven endophyte conidial suspensions of 1×10^8^ conidia ml^−1^ for 10 hours. Control plants were dipped in sterile distilled water containing 0.05% Triton X-100. The inoculated seedlings were transplanted in pots containing sterile soil as described earlier. The experimental design was a completely randomized block design (CRBD) with four replicates. The plants were maintained under similar conditions as those inoculated through the seeds.

#### Assessment of colonization

To determine colonization by inoculated fungal isolates, onion plants were carefully uprooted from the pots after 50 and 70 days for inoculated seeds and seedlings, respectively. Plants were then washed gently with water. Leaves, stems and roots were separated from each plant. Sections of leaves were sampled from the middle and outer leaves of the plant while the whole lengths of stems and roots were used for sampling. The sampled plant parts were then surface-sterilized by dipping them in 70% ethanol and then immersing in 2% NaOCl for 2 and 3 min, respectively, and rinsed three times using sterile distilled water. The final rinse water was plated on PDA to confirm elimination of epiphytic microorganisms as described earlier. The surface-sterilized plant parts were then aseptically cut into 1 cm lengths under a laminar flow hood. Five randomly selected pieces were placed in uniform distribution on PDA plates amended with antibiotics (tetracycline and streptomycin sulfate salt at 0.05%) [31] and incubated in the dark at 25°C for 10 days, after which the presence of fungal growth was observed. Positive colonization was scored by counting the number of pieces of the different plant parts with growth of inoculated endophyte. To confirm whether the growing endophytes were the ones initially inoculated; slides prepared from the mother plates were used for comparison and morphological identification.

### Effect of endophytically colonized onion plants on proportion of thrips observed on plants, feeding punctures and oviposition

Seed inoculation technique was found to be effective for colonization and was therefore adopted for this study. Seeds inoculated with all fungal isolates and a control were transplanted in smaller pots (diameter 8 cm) with one plant per pot until 3- to 5-leaf stage before being used in the experiment. Plants with four fully grown leaves were exposed to one-day-old (presumably mated) adult female thrips (10 individuals) for 72 h in Plexiglas cages (30×30×25 cm) and were maintained at 26±1°C, 50–70% RH and 12L: 12D photoperiod. A total of four cages were used for each treatment. After 72 h, all adult thrips observed on the plants were recorded. The individual plants were cut and placed in labeled polythene paper bags for later quantification of thrips feeding and oviposition activities. Two leaves from each plant were cut into three sections of 4 cm each, from the base, middle and tip of the leaf. The number of feeding punctures was counted under a stereomicroscope and recorded. The sections were stained in boiling lactophenol-acid fuchsin solution [32] for 30–40 mins. After staining, the leaves were placed in 90 mm Petri dishes for 1 h before being destained. Destaining was done by immersing the leaves in warm water for three minutes after which the eggs were counted under a stereomicroscope. Treatments were randomized in complete block design and the experiment replicated four times. Verification of colonization of onions by the endophytes was performed at the end of the experiment.

### Data analysis

Binary data on colonization (presence or absence) were fitted in a generalized linear mixed model assuming binomial distribution error and logit using package *lme*4 [33] in R 2.15.2 statistical software [34]. Treatments were considered as fixed effects and the plant pieces nested within the plant as random effects. The extent of fungal colonization (%) of host plant parts was calculated as detailed below.
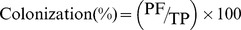
where – PF – Number of pieces exhibiting fungal growth, TP – Total number of pieces plated out.

The numbers of thrips observed on the onion plants were recorded for all treatments and replicates. Analysis was performed using logistic regression model which was fitted to the data on proportion of thrips recovered 72 h post-exposure using package HSAUR [35] in R 2.15.2. The number of feeding punctures on each leaf section were determined and summed up per plant before staining the leaves for eggs count. All count data on feeding and oviposition of *T. tabaci* were checked for normality and homogeneity of variance using Shapiro-Wilk and Levene tests, respectively, before analysis by negative binomial regression using R 2.15.2 [34] with package MASS [36]. The negative binomial distribution was chosen, based on its biological appropriateness in handling overdispersion in count data. P-values of <0.05 were considered as significant.

## Results

### Seed and seedling colonization of onion plants by fungal endophytes

**Figure 1:**
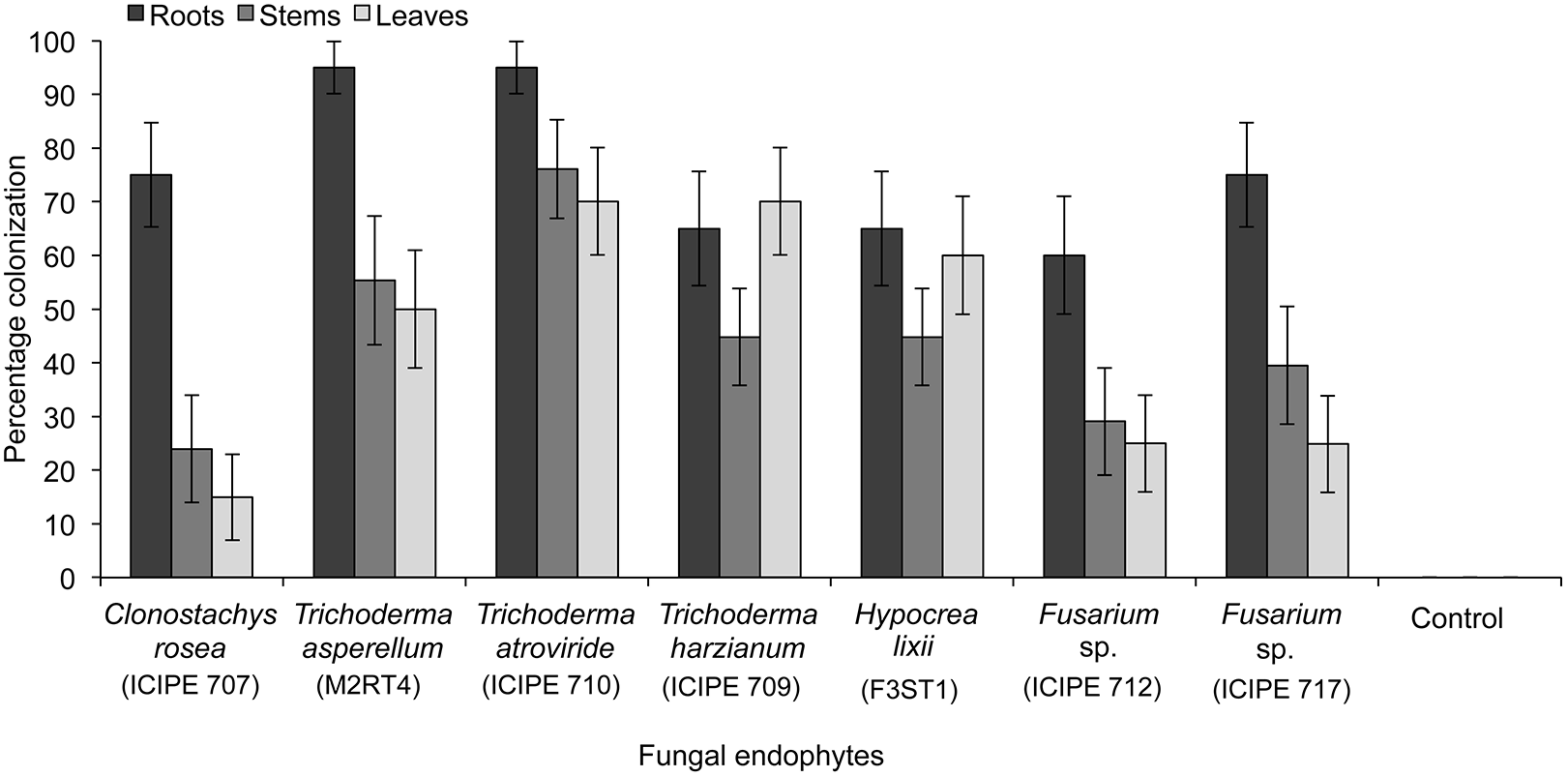
Endophytic colonization of onion seeds. Percentage colonization of onion plant parts (root, stem and leaves) by different fungal endophytes through seed inoculation. Data are percentage mean ± SE. (P≤0.05).

**Figure 2:**
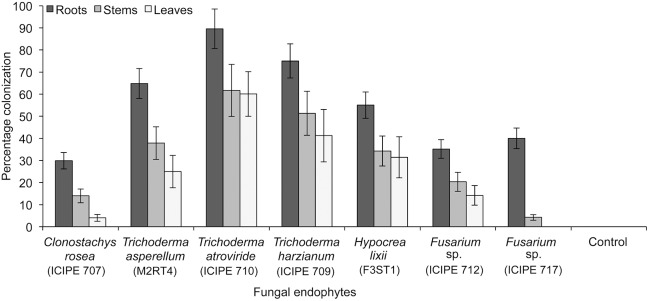
Endophytic colonization of onion seedlings. Percentage colonization of onion plant parts (root, stem and leaves) by different fungal endophytes through seedling inoculation. Data are mean ± SE. (P≤0.05).

The viability tests yielded >90% germination of conidia, for all the isolates. Since the final rinse water did not show any sign of fungal growth on the media, it was concluded that the surface sterilization technique used was effective. All the tested fungal isolates were able to colonize onion plants following seed or seedling inoculation (Figures 1, 2). However, the extent of colonization of the different plant parts depended on the inoculation method and the fungal isolate. Seed inoculation resulted in 1.47 times higher mean percentage post-inoculation recovery of all the endophytes tested as compared to seedling inoculation (F = 11.13; df = 1, 3; p = 0.002). For example, mean colonization of roots by *C. rosea* ICIPE 707 isolate was 75.00±9.7% through seed inoculation and 29.85±3.7% through seedling inoculation. Seed inoculation method resulted in higher mean post-inoculation recovery of all the endophytes tested for roots, stems and leaves (76.06±4.1%, 44.24±3.6% and 44.73±5.4%), respectively (Figure 1). On the other hand, seedling inoculation recorded 55.62±4.5%, 31.75±5.8% and 24.65±6.8% for roots, stems and leaves, respectively (Figure 2).

### Effect of endophytically colonized onion plants on proportion of thrips observed on plants, feeding punctures and oviposition

**Figure 3:**
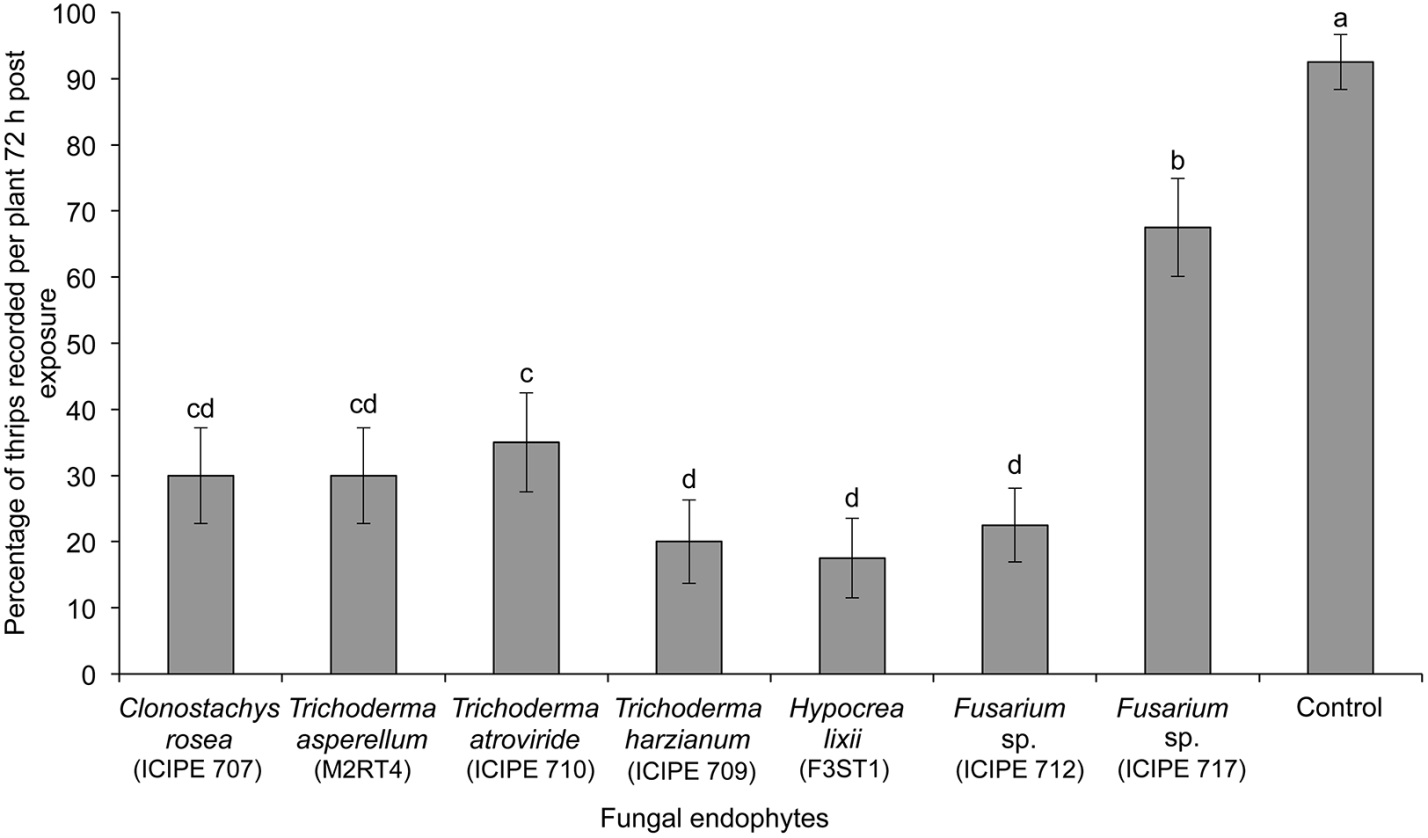
Effect of endophytically colonized onion plants on proportion of adult *Thrips tabaci*. An evaluation of fungal endophytes for their effect on proportion of thrips settling on inoculated onion plants after 72 h. Bars indicate means ± SE at 95% CI. Means followed by the same letter indicate no significant differences between treatments.

**Figure 4:**
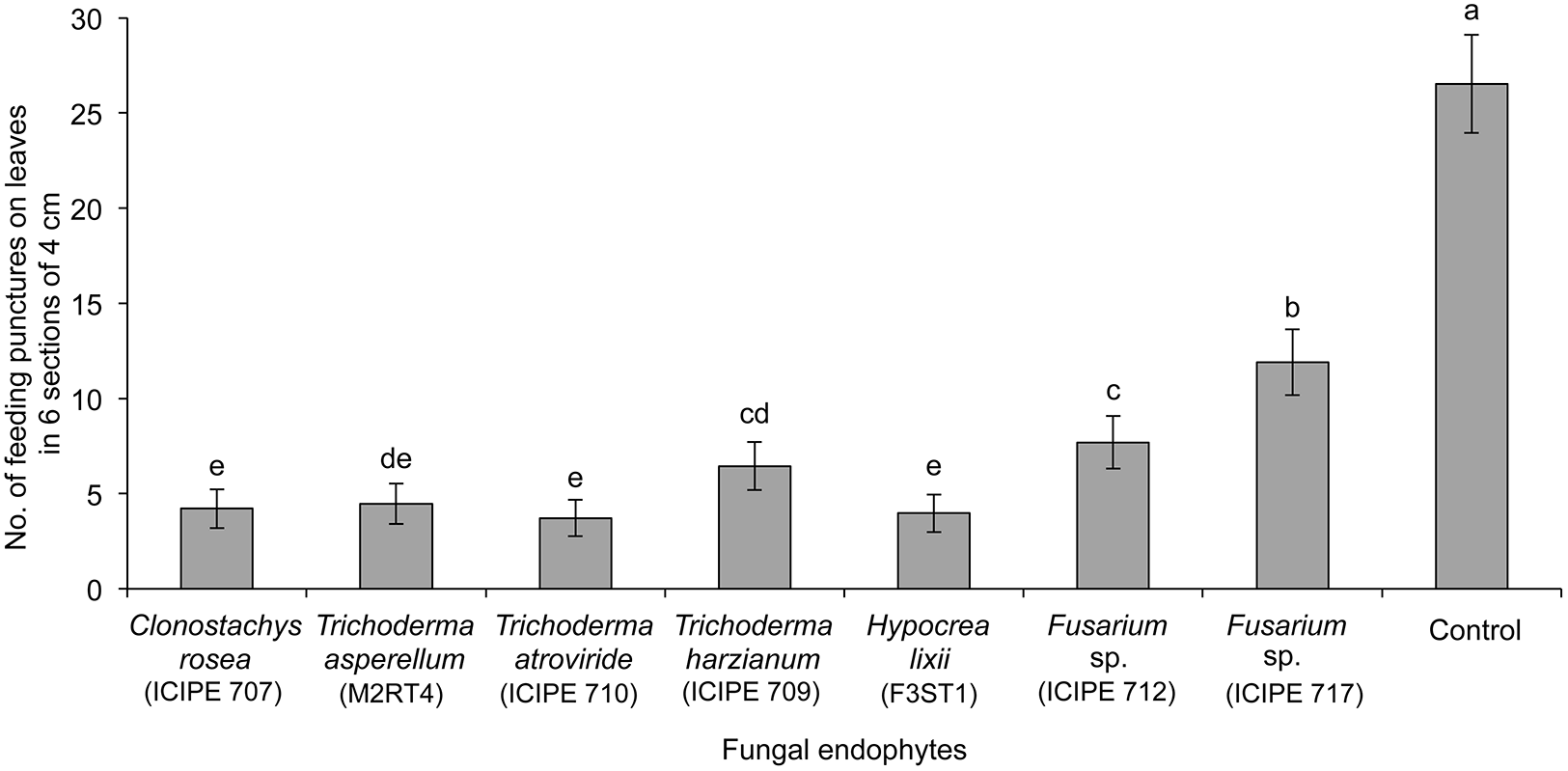
Effect of endophytically colonized onion plants on feeding punctures by adult *Thrips tabaci*. The figure quantifies mean feeding activity by *Thrips tabaci* exposed for 72 h on onion plants inoculated with different fungal endophytes. Bars indicate means ± SE at 95% CI. Means followed by the same letter indicate no significant differences between treatments.

The treatments had a significant effect on the proportion of thrips observed on the onion plants 72 h post-exposure (χ^2^ = 87.79, df = 7, p<0.001) (Figure 3). Overall *Hypocrea lixii* outperformed all the other treatments in affecting the proportion of thrips on the plants. Fewer thrips were observed on plants inoculated with *C. rosea* ICIPE 707, *T. asperellum* M2RT4, *T. atroviride* ICIPE 710, *T. harzianum* ICIPE 709, *H. lixii* F3ST1 and *Fusarium sp.* ICIPE 712 isolates as compared to those inoculated with *Fusarium* sp. ICIPE 717 and the control treatments (Figure 3). The number of feeding punctures by *T. tabaci* was significantly lower in all the endophyte-inoculated plants as compared to the control treatment (F = 22.71; df = 7, 21; p<0.001; n = 4) (Figure 4). Plants colonized by isolates *C. rosea* ICIPE 707, *T. asperellum* M2RT4, *T. atroviride* ICIPE 710, and *H. lixii* F3ST1 had significantly lower number of feeding punctures as compared to the other treatments (Figure 4).

**Figure 5:**
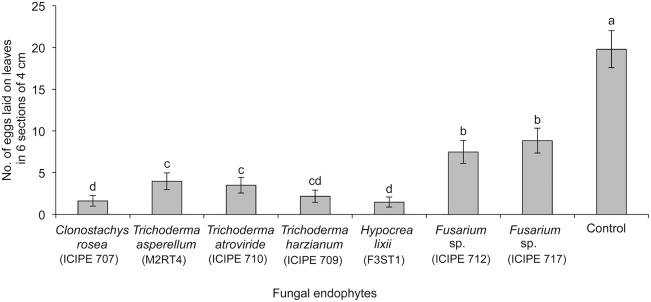
Effect of endophytically colonized onion plants on oviposition by adult *Thrips tabaci*. The figure shows the mean number of eggs laid by *Thrips tabaci* on onion plants endophytically colonized by different fungal isolates. Bars indicate means ± SE at 95% CI. Means followed by the same letter indicate no significant differences between treatments.

Highest number of eggs (18.6±2.2) was oviposited by *T. tabaci* in the control plants than in all other endophytically colonized plants (F = 16.75; df = 7, 21; p<0.001) (Figure 5). Among the isolates tested, the lowest numbers of eggs were laid by *T. tabaci* on *H. lixii* F3ST1 and *C. rosea* ICIPE 707 inoculated plants. Plants inoculated with *T. asperellum* M2RT4 and *T. atroviride* ICIPE 710 isolates were equally effective in their capacity to reduce egg laying by *T. tabaci*. *Fusarium* sp. ICIPE 717 colonized plants showed about 6 times higher number of eggs as compared to *H. lixii* F3ST1 (Figure 5).

## Discussion

Plant colonization depended on inoculation methods. For instance, seed inoculation method resulted in superior colonization of onion plants as compared to the seedling inoculation. The difference in colonization between the two may be explained in part by a reduced capacity of uninoculated seedlings to enhance endophyte proliferation due to transplantation shock [37]. Moreover, endophyte inoculation at seed stage could have the advantage of colonizing both seed radical and the plumule, which are close to one another in the seed. Tefera and Vidal [38] reported that seed inoculation of sorghum plants with *B. bassiana* resulted to good endophyte colonization in vermiculate and sterile soil substrates. Seed inoculation could be advantageous in terms of low inoculums requirement as compared to augmentative sprays [23]. Further seed treatment could provide opportunities for endophytic fungi colonization at the young seedling stage for early protection and enhanced seedling health. Backman and Sikora [39] outlined that, integrated pest management on seeds reduces costs and environmental impact, while allowing the biological agent to build up momentum for biological control. Posada et al. [40] found that direct injection of *B. bassiana* conidial suspensions had the highest post-inoculation recovery in coffee seedlings than foliar sprays, stem injections, or soil drenches. Our results show that there were differences in the level of colonization of different plant parts by fungal isolates. For instance, roots sections had higher colonization as compared to stems and leaves. These differences could be due to tissue specificity exhibited by endophytic fungi and their adaptation to particular physiological conditions of the plants [41]. Similar results were reported on French beans and Faba beans [18] and coffee [40].

Among the endophytes that colonized onion plants, *C. rosea* ICIPE 707, *H. lixii* F3ST1, *T. harzianum* ICIPE 709, *T. atroviride* ICIPE 710, and *T. asperellum* M2RT4 had significantly low proportion of thrips, number of feeding punctures and eggs. However, isolate *H. lixii* F3ST1 had the highest overall negative impact on *T. tabaci*. Lately, the impacts of fungal endophytes on suppression of different insect groups in different host plants are receiving increased attention [16], [18], [42]. The negative effect on the proportion of thrips on the endophyte-colonized plants as compared to the control could have been responsible for reduced feeding and oviposition. For instance, Akutse et al. [18] reported that Faba beans colonized endophytically by fungal endophytes of the genera *Hypocrea* and *Beauveria* had significant negative effects on leafminer, *Liriomyza huidobrensis* (Blanchard) fitness, impacting on mortality, oviposition, emergence and longevity of the pest. Cherry et al. [16] found a reduced number of *Sesamia calamistis* (Hampson) in *B. bassiana*-inoculated plants compared to non-inoculated plants. Thrips are able to distinguish among plants as suitable for feeding and/or oviposition sites to ensure fitness of their progeny [43]. *Thrips tabaci* is a key vector of *Iris yellow spot virus* (IYSV) in Kenya [44], [45] and the thrips densities are positively associated with IYSV incidence [46], [47], [48]. Hence, the reduced feeding by the thrips on endophyte-colonized plants could potentially reduce the transmission of IYSV in onions. Moreover, fungal endophytes can decrease plant virus infections in plants as reported in meadow ryegrass with the *Barley yellow dwarf virus* (BYDV) [49]. The broad array of endophyte induced defence mechanisms in plants against insect pests such as production of toxic or distasteful chemicals [50] and pathogenic interaction to insects [51] could decrease insect fitness [52], a phenomenon that needs to be further investigated.

In the present study, dead insects did not present any signs of mycosis. Previous studies have also revealed that dead insects recovered from endophytically-colonized plants exhibit no signs of fungal infection [16], [18]. The influence of endophytes colonizing onions on thrips biology in terms of observable proportion, feeding and oviposition in the present study are in accordance with the findings by Cherry et al. [16] and Bittleston et al. [53] on reduced feeding and by Akutse et al. [18] on oviposition with other endophytes and pests. The reduced feeding and oviposition could have been a result of either reduced survival of thrips or antixenotic repellence of thrips, phenomena that warrant further studies to unravel the underlying mechanisms such as possible release of metabolites and/or volatiles which could have effects on thrips.

Isolates *C. rosea* ICIPE 707, *H. lixii* F3ST1, *T. harzianum* ICIPE 709, *T. atroviride* ICIPE 710, and *T. asperellum* M2RT4 effectively colonized the various plant parts of onion as compared to the *Fusarium* isolates. Consequently, isolate *H. lixii* F3ST1 had the most antagonistic impact on onion thrips and it could be used to develop alternative and ecologically safe management strategy for onion thrips. We conclude that, onions can be successfully inoculated especially through seeds, with different fungal endophytes. However, further studies are warranted to determine the persistence of tested endophytes in the colonized plants under natural conditions and investigate potential for vertical transmission of endophytes. Additionally, being the first report of antagonistic activity of endophytes colonizing onion against *T. tabaci*, it would be crucial to determine the underlying mechanisms of such multi-trophic interactions.
